# *Gymnosporangium yamadae* Effector GyHRb12 Targets the Host Ribosomal Protein MdRPS20 to Enhance Translation and Suppress Immunity of Apple Leaves

**DOI:** 10.3390/ijms27072970

**Published:** 2026-03-25

**Authors:** Chuxing Li, Chenxi Shao, Yingmei Liang

**Affiliations:** 1 The Key Laboratory for Silviculture and Conservation of Ministry of Education, Beijing Forestry University, Beijing 100083, China; 2 Museum of Beijing Forestry University, Beijing Forestry University, Beijing 100083, China

**Keywords:** rust fungi, effector protein, immune-suppressive factor, ribosomal subunit, *Malus domestica*

## Abstract

The apple rust fungus *Gymnosporangium yamadae* (*G. yamadae*) secretes effector proteins into host apple leaf cells to facilitate parasitism. Among these, the candidate effector GyHRb12 was found to localize to the nucleus upon transient expression in *Nicotiana benthamiana* leaf cells, although its functional role remained unclear. Subsequent investigations demonstrated that overexpression of GyHRb12 protein decreases plant cell resistance and attenuates the transcription of multiple antifungal-related genes. Using a yeast two-hybrid screen, MdRPS20, a component of the 30S ribosomal subunit, was identified as an interactor of GyHRb12. Proteomic analysis revealed that GyHRb12 modulates the expression of proteins involved in protein translation processes, which may be mediated by changes in ribosomal abundance. Notably, mutating the 14th amino acid in MdRPS20 disrupted its interaction with GyHRb12, underscoring the critical role of this residue in effector recognition and subsequent suppression of host immunity. Collectively, these findings demonstrate that *G. yamadae* employs a nuclear-localized effector to target a ribosomal subunit protein, thereby reprogramming host translation activity and suppressing host immunity.

## 1. Introduction

Plants in natural environments face constant attack by diverse pathogens. As obligate biotrophic pathogens, rust fungi have evolved to suppress host immune responses while maintaining host viability to successfully complete their life cycle. During infection, haustoria penetrate host cells to absorb nutrients [1,2], and secrete effector proteins to suppress or interfere with host immunity [3,4,5].

Protein biosynthesis is a critical process frequently targeted by pathogen effectors. In filamentous fungi, numerous effector proteins have been characterized that modulate key stages of gene expression, such as transcription (e.g., Mlp124478 [6,7], PstGTA1 [8]), alternative splicing (e.g., PstA-23 [9]; PsAvr3c [10,11]), translation (e.g., Pi23226 [12]; CSEP0064/BEC1054 [13]; Zt6 [14]), and post-translational modifications (e.g., Hasp98 [15]) to suppress host immunity. By contrast, rust fungal effectors that specifically target translation remain largely uncharacterized. Nevertheless, transcriptomic and proteomic analyses have revealed significant alterations in protein synthesis pathways, including ribosome assembly and endoplasmic reticulum protein processing, during resistance responses, highlighting the importance of coordinated protein regulation in host defense [16,17]. This observed enrichment of protein synthesis-related pathways is thought to reflect directional selection for disease resistance [18,19], raising the possibility that the effectors of rust fungi may manipulate host protein synthesis to promote infection.

Ribosomes are essential for protein translation [20]. The small 30S subunit incorporates ribosomal protein S20 (RPS20), a critical component required for mRNA decoding [21,22,23]. In plants, RPS20 is indispensable for normal growth and development. Disruption of RPS20 in *Oryza sativa* impairs chloroplast development, profoundly suppresses transcript levels of nuclear genes governing chlorophyll biosynthesis and chloroplast development are strongly affected in rice cells [24]. Similarly, *Arabidopsis thaliana* (*A. thaliana*) lacking RPS20 exhibits compromised basal ribosome activity, altered cell division, and embryonic arrest prior to the heart stage [25]. Beyond their canonical roles in protein synthesis, accumulating evidence indicates that ribosomes and ribosomal proteins actively participate in the regulation of plant immunity. For instance, transgenic StoL13a plants exhibited differential upregulation of five defense genes following *Verticillium dahliae* infection, suggesting its role in the defense response and reduced ROS accumulation, thereby enhancing oxidative stress tolerance [26]. Similarly, overexpression of L16D in *A. thaliana* induced the expression of many pathogen resistance-related genes while repressing growth-related gene expression, highlighting a regulatory trade-off between growth and defense mediated by ribosomal components [27]. Collectively, these findings indicate that ribosomes function not only as translational machines but also as key regulatory nodes coordinating plant growth and immune responses. However, whether rust fungi employ effectors to manipulate ribosome-mediated defense responses remains unresolved.

The obligate biotrophic phytopathogen *Gymnosporangium yamadae* (*G. yamadae*) [28] causes rust disease in *Malus* plants, leading to serious yield losses [29,30]. Its telial host, juniper, harbors the related species *G. asiaticum*, whose aeciospores or pycniospores parasitize *Pyrus* spp. plants [28] rather than *Malus* spp. Comparative transcriptomic analysis revealed that *G. asiaticum* exhibits heightened protein biosynthesis activity compared to *G. yamadae*, although no significant differences in gene enrichment were observed between them [31]. Paradoxically, despite typical host selectivity among these species, haustorium-associated biological processes are conserved, leaving the host selection mechanism unexplained. Consequently, many effector proteins are secreted into host cells in order to accelerate infection through haustoria. Differences in effector protein function could explain their host selection mechanism. Numerous candidate effector proteins are secreted during *Malus domestica* (*M. domestica*) infection [31,32,33,34], implying a complex interaction mechanism between *Gymnosporangium* and their hosts. For example, GyBarwin55 and GyRlpA22 depend on their NLS sequences for their pathological functions [35]. Nevertheless, target identification remains limited. Gy29 interacts with MdGDPD6 and MdL-ASNase to regulate the metabolism of phospholipids and proteins in apple leaves [36]. To date, only a few effectors have been functionally characterized, and many secreted proteins remain poorly understood.

Building on previous findings that the *G. yamadae* haustoria-secreted effector GyH12 localizes to the nucleus of *N. benthamiana* cells, this study further validates its pathological role as a potent suppressor of host immune responses. Because this effector protein promotes virulence through association with a ribosomal protein, the *M. domestica* 30S ribosomal protein S20 (MdRPS20), it is redesignated as GyHRb12 (*G. yamadae* Haustoria secreted Ribosomal binding effector protein No. 12). Our results reveal a previously unrecognized mechanism by which a rust fungal effector targets host ribosome-associated processes. Through this interaction, GyHRb12 appears to coordinate host cellular viability and immune suppression. Both processes are essential for sustained nutrient acquisition by obligate biotrophic pathogens. Notably, the requirement for a specific recognition site in MdRPS20 suggests that precise effector–host protein interactions may underlie host specificity and selection. Collectively, these findings indicate that rust fungal effectors can exploit host ribosomal components to balance nutrient-dependent host viability with immune suppression, thereby facilitating successful colonization and providing new insights into host selection mechanisms during infection.

## 2. Results

### 2.1. GyHRb12 Suppresses the Immunity of M. domestica

**Figure 1:**
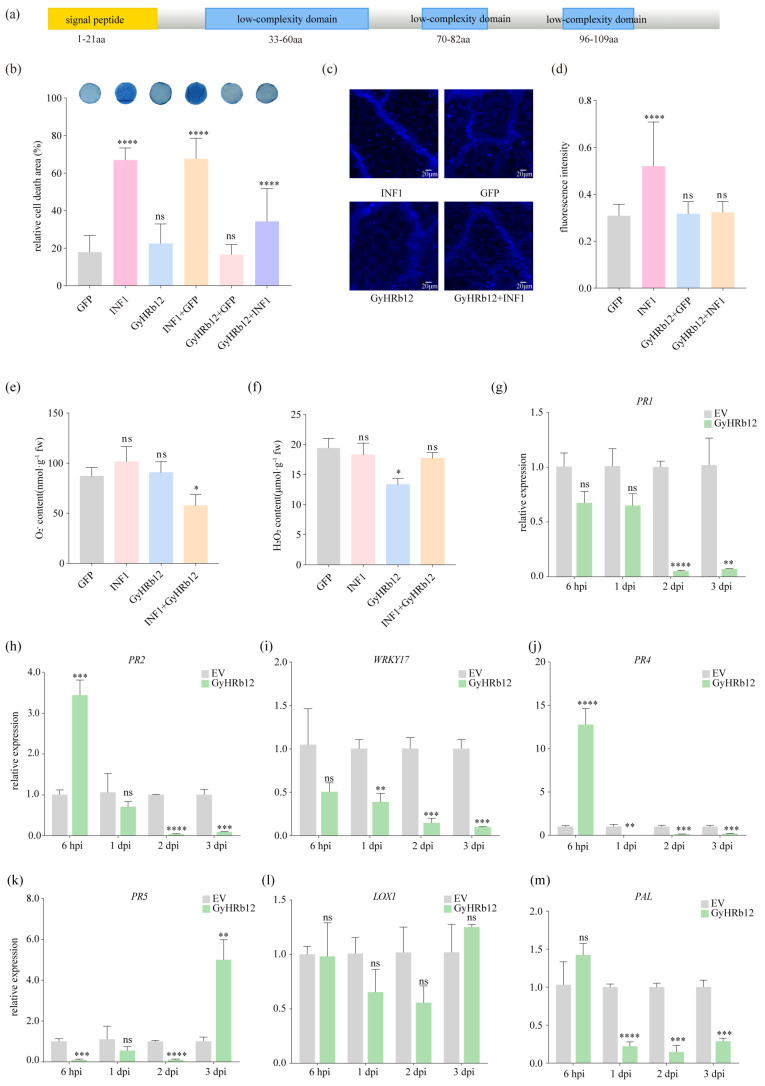
Sequence alignment results of GyHRb12 gene and its pathological function validation. (**a**) The structure of GyHRb12 protein was predicted using the NCBI database and MEME with default parameters, and then manually visualized. (**b**) GyHRb12 suppresses the programmed cell death (PCD) induced by INF1 in *Malus domestica* (*M. domestica*) leaves. The leaves were infiltrated with *Agrobacterium tumefaciens* (*A. tumefaciens*) GV3101 carrying GFP, INF1 or GyHRb12. (**c**,**d**) GyHRb12 suppresses the callose deposition induced by INF1 in *M. domestica* leaves. (**e**,**f**) H_2_O_2_ and O_2_^−^ detected after 24 h of *A. tumefaciens* infiltration. Values represent the mean ± SEM from three biological replicates. Significant differences compared with GFP in (**b**–**f**) were determined using one-way ANOVA followed by a paired *t*-test for post hoc comparisons. (**g**–**m**) qRT-PCR analysis of resistance-related marker genes in *M. domestica* leaves at 6 hpi to 3 dpi after agro-infiltration empty vector (EV) or GyHRb12-PBI121. At each time point, expression levels were normalized to the corresponding EV control group. Significance was determined by multiple *t*-tests with Benjamini-Krieger-Yekutieli two-stage linear step-up adjustment (Q < 0.05). Data are presented as the mean ± SEM from three biological replicates, each with three technical replicates.* *p* < 0.05, ** *p* < 0.01, *** *p* < 0.001, **** *p* < 0.0001, and ns was not significant.

Identified in prior research, the new haustoria-secreted effector GyHRb12 remained functionally uncharacterized during *G. yamadae* infection. The GyHRb12 coding sequence (CDS) was 375 bp, encoded a 125-amino acid (aa) protein featuring a signal peptide (1–21aa) and three low-complexity regions (Figure 1a). In contrast to typical cysteine-rich effectors, GyHRb12 was characterized as a serine-rich effector, with serine residues accounting for 20.16% of the mature protein. NCBI BLAST analysis (https://blast.ncbi.nlm.nih.gov) indicated no homologs of GyHRb12 in publicly available genomes, and MEME analysis detected no conserved motifs in its protein sequence. Together with bioinformatic predictions and our previous findings, these results suggested that GyHRb12 is a specific effector of *G. yamadae*.

To further confirm the immune function of GyHRb12 in apple leaves, the effector was transiently expressed in apple leaf discs through vacuum infiltration using *Agrobacterium tumefaciens* (*A. tumefaciens*) GV3101 strain. Western blot assays confirmed expression of the mCherry-tagged GyHRb12 (Figure S1). Trypan blue staining revealed significantly reduced cell death in discs co-infiltrated with GyHRb12 and INF1 compared to INF1-PGR106 alone (Figure 1b), indicating that immune suppression occurred in GyHRb12-infiltrated apple leaves. To examine whether GyHRb12 suppresses host defense responses, callose deposition and ROS bursting were measured after agroinfiltration of apple leaves. As expected, GyHRb12 alone did not induce callose deposition and significantly reduced the callose deposition which was induced by INF1 (Figure 1c,d). Additionally, the levels of H_2_O_2_ and O_2_^−^ showed that GyHRb12 reduced the production of O_2_^−^ (Figure 1e,f). At the transcriptional level, the expression levels of genes involved in resistance were analyzed. Accordingly, the expression of six resistance-associated genes, *PR4*, *PR2*, *PR5*, *PR1*, *PAL*, and *WRKY17*, was repressed during periods of high GyHRb12 expression, with the exception of *LOX1* (Figure 1g–m). The relative expression of GyHRb12 reached its highest level on the second day of transient transformation (Figure S2).

Collectively, these results showed that GyHRb12 functions as an immune-suppressive effector between plants and pathogens, although its inhibitory effect appears to be partial.

### 2.2. GyHRb12 Activates the Synthesis of Translation-Associated Proteins in Apple Leaves

**Figure 2:**
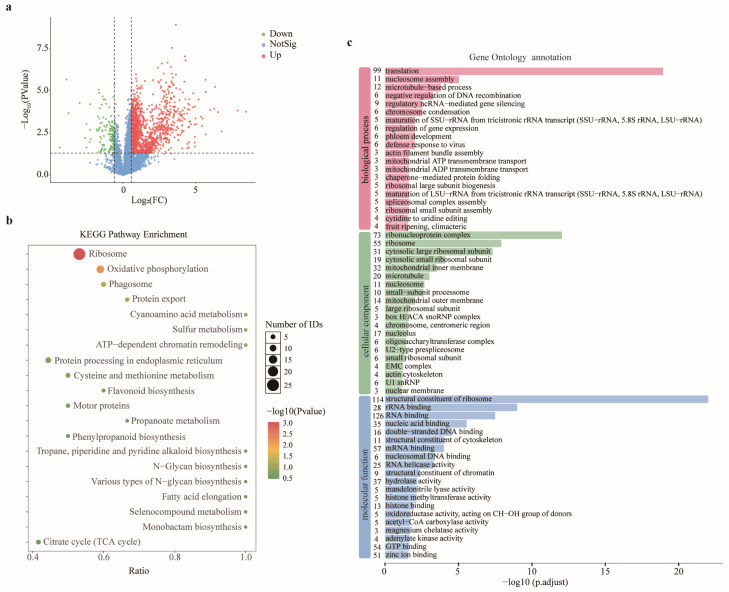
Proteomic changes in apple leaves under the influence of GyHRb12. (**a**) Volcano plots of differentially expressed proteins (DEPs) identified between GyHRb12-expressing and empty vector (EV) control apple leaves by DIA-based LC-MS analysis. The green dots show down-regulated proteins (log2(FC) < log2(1/1.5), *p*-value < 0.05) and the red dots show up-regulated proteins (log2(FC) > log2(1.5), *p*-value < 0.05). The y-axis represents −log10(*p*-value), whereas the x-axis represents log2(FC). Dashed lines indicate the thresholds for fold change and statistical significance. (**b**) The Kyoto Encyclopedia of Genes and Genomes (KEGG) pathway enrichment for DEPs. The size of each dot represents the number of proteins, and the color indicates the *p*-value. (**c**) Gene Ontology (GO) analysis of the differentially expressed proteins between EV and GyHRb12 overexpressing apple leaves (*p* < 0.05). The results were categorized into three groups: Biological process, Cellular component and Molecular function.

In order to explore the role played by GyHRb12 in the host, data-independent acquisition (DIA) mass spectrometry (MS) was used to compare the proteomes of the GyHRb12 transient expression and empty vector control (EV) treatment groups at 3 dpi. In total, 6284 proteins were identified by LC–MS, with 4536 and 5927 proteins detected in the EV and GyHRb12 groups, respectively (Table S2). Differential expression analysis (|log2(FC)| ≥ log2(1.5), *p* < 0.05) identified 1694 differentially expressed proteins (DEPs), comprising 1569 up-regulated and 125 down-regulated proteins (Figure 2a). Most of the highly expressed proteins displayed marked fold changes (Table S2), exemplified by DVH24_041886 (an RRM domain-containing protein, FC = 357.5) and DVH24_006233 (an ABC transporter domain-containing protein, FC = 239.7). Among 67 identified defense-related proteins, 21 showed significant differential expression (Table S3), including the up-regulation of a peroxidase (DVH24_031815), further corroborating the modulation of ROS metabolism in apple leaves.

The Kyoto Encyclopedia of Genes and Genomes (KEGG) pathway enrichment analysis of these DEPs revealed predominant enrichment of up-regulated proteins in ‘ribosome’ and ‘oxidative phosphorylation’ pathways (Figure 2b), although not all ribosomal proteins exhibited significant alterations (Table S4). Correspondingly, Gene Ontology (GO) enrichment analysis (Figure 2c) indicated that DEPs were primarily linked to molecular functions including ‘structural constituent of ribosome’ and ‘RNA binding’. Within biological processes, up-regulated proteins were mainly associated with ‘translation’, while in cellular components they showed enrichment in ‘ribonucleoprotein complexes’ and ‘ribosome’, collectively highlighting that GyHRb12 activated ribosomal protein expression in apple leaves, thereby potentially increasing cellular energy expenditure and protein synthesis activity.

### 2.3. GyHRb12 Targets M. domestica 30S Ribosomal Protein S20

**Figure 3:**
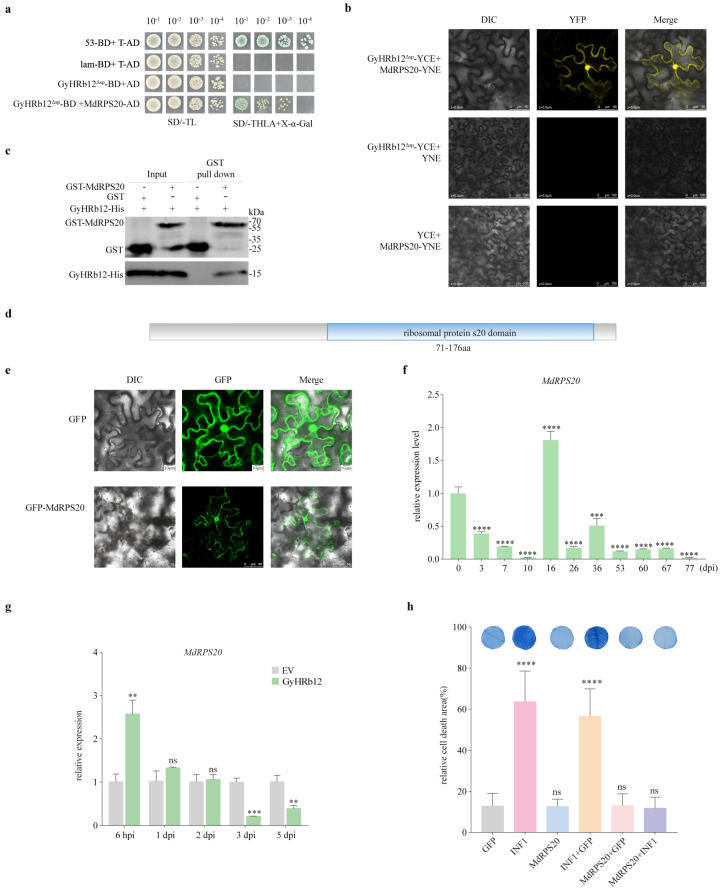
Interaction between *Gymnosporangium yamadae* (*G. yamadae*) effector GyHRb12 and *M. domestica* protein MdRPS20. (**a**) Yeast two-hybrid (Y2H) analysis of the interaction between GyHRb12 and MdRPS20. Yeast strains co-carrying constructs GyHRb12(ΔSP)-pGBKT7 (BD) and MdRPS20-pGADT7 (AD) were grown on SD media-TL and SD-THLA with X-α-gal. 53-BD/T-AD was used as the positive control, and GyHRb12(ΔSP)-BD/AD and lam-BD/T-AD were used as the negative controls. (**b**) Bimolecular fluorescent complementation (BiFC) assay of the interaction between GyHRb12 and MdRPS20. The GyHRb12-YCE/YFP and YCE/MdRPS20-YNE were used as negative controls. (**c**) GST pull-down assay of the interaction between GyHRb12 and MdRPS20. Glutathione agarose purified GST-MdRPS20 or GST was incubated with an equal amount of *Escherichia coli* lysates containing His-GyHRb12, followed by the addition of glutathione eluent. The pulled down proteins were then detected by Western blotting using anti-GST and anti-His antibodies. (**d**) The structure of MdRPS20 protein was analyzed using the NCBI and manually illustrated. (**e**) Subcellular localization of MdRPS20 protein fused with GFP in overexpressing *Nicotiana benthamiana* leaves. GFP fluorescence was observed under a fluorescence microscope. The native control was an empty pBinGFP vector. (**f**) The relative expression of MdRPS20 during *G. yamadae* infection detected by qRT-PCR. Data are presented as the mean ± SEM from three independent biological replicates, each measured in triplicate technical replicates. Statistical analysis relative to the expression level at 0 dpi was performed using one-way ANOVA followed by Benjamini–Krieger–Yekutieli two-stage linear step-up for multiple comparisons, with a significance threshold set at *p* < 0.05. (**g**) Expression of MdRPS20 in transiently expressing GyHRb12 apple leaves detected by qRT-PCR. At each time point, expression levels were normalized to the corresponding EV control group. Statistical significance was determined using multiple *t*-tests with the Benjamini-Krieger-Yekutieli two-stage linear step-up procedure (FDR < 0.05). Data are presented as the mean ± SEM from three biological replicates, each with three technical replicates. (**h**) Transient overexpression of MdRPS20 suppressed PCD in apple leaves. Significant differences compared to the GFP protein were determined using one-way ANOVA. Asterisks indicate significant differences compared to the GFP control group (** *p* < 0.01, *** *p* < 0.001, **** *p* < 0.0001; ns, not significant).

Based on the above results, GyHRb12 was most likely involved in host ribosome or translation-related function. To identify host targets of the effector GyHRb12, a yeast two-hybrid (Y2H) cDNA library constructed from *G. yamadae*-infected apple leaves was screened using GyHRb12 without its signal peptide (GyHRb12^Δsp^) cloned into the pGBKT7 vector as bait. After SD/-Leu-Trp and SD/-Leu-Trp-His-Ade (with X-α-gal) media screens, 36 possible candidate targets were obtained. Taking into account the nuclear localization of GyHRb12 and that it regulated the translation in apple leaves, 11 candidate target proteins were prioritized for validation (Table S1). Among these, the 30S ribosomal protein S20 (NCBI Reference Sequence: XM_008387984.3; MdRPS20) was captured. To confirm the preliminary Y2H results, the full-length MdRPS20 was cloned into the pGADT7 vector as prey. Y2H assays showed that GyHRb12^Δsp^ was capable of interacting with MdRPS20 (Figure 3a). This interaction was further supported by a bimolecular fluorescence complementation assay, wherein co-expression of MdRPS20-YNE and GyHRb12^Δsp^-YCE resulted in detectable fluorescence, while control combinations with empty vectors showed no signal, thereby validating the interaction in vivo (Figure 3b). Meanwhile, a pull-down assay using recombinant proteins expressed in *Escherichia coli* (*E. coli*) BL21 demonstrated that GyHRb12^Δsp^-His was specifically retained by MdRPS20-GST bound to glutathione resins, but not by GST alone (Figure 3c). Together, these results establish that GyHRb12 interacts with MdRPS20 both in vivo and in vitro.

Sequence analysis revealed that the CDS of MdRPS20 is 555 bp, encoding a 185 aa protein containing a ribosomal S20 domain (71–176aa) (Figure 3d). According to InterPro annotations, MdRPS20 participated in the translation process (GO:0006412) and possessed RNA-binding activity (GO:0003723). LOCALIZER predicted that MdRPS20 localized to both the chloroplast and nucleus and contained a nuclear localization signal (NLS). In addition, a chloroplast transit peptide was predicted in amino acids 1–70 by TargetP 2.0 (Figure S3). To identify its precise localization in planta, MdRPS20 was fused to the N-terminus of GFP (pBinGFP2:MdRPS20), with the empty pBinGFP2 vector serving as a negative control. Confocal laser scanning microscopy images showed that GFP-MdRPS20 generated a strong fluorescence in the nucleus and cytosol (Figure 3e). These results showed that MdRPS20 is a ribosomal protein, and localizes in nucleus and cytosol.

To clarify the expression pattern of MdRPS20 during apple rust disease development, apple leaves were collected at different stages following infection with *G. yamadae* for total RNA extraction. Samples collected at 0 dpi were designated as the reference time point in this study. qRT–PCR analysis showed that MdRPS20 transcript levels were significantly decreased during the haustorium formation stage of *G. yamadae* infection (Figure 3f). To determine whether MdRPS20 was subject to regulation by GyHRb12, MdRPS20 transcript levels were quantified in apple leaves within 5 days post-infiltration of GyHRb12. qRT–PCR analysis showed that the transcript level of MdRPS20 increased initially and subsequently decreased during infection (Figure 3g), whereas the proteomic analysis revealed a significant enrichment of the MdRPS20 protein (FC = 1.85, *p* < 0.01). Given the potential role of ribosomes as defense components during pathogens–host interaction and the defense role of MdRPS20 in immunity, its effect on programmed cell death (PCD) was examined in apple leaves. Using the previously described GV3101 vacuum infiltration method, the expression of mCherry-tagged MdRPS20 was confirmed by Western blot (Figure S1). Transient overexpression of MdRPS20 significantly suppressed PCD (Figure 3h), mirroring the phenotype induced by GyHRb12, which implies that MdRPS20 might be induced by GyHRb12 to attenuate host defense responses.

### 2.4. Leucine Is a Critical Recognition Site of GyHRb12 for MdRPS20

**Figure 4:**
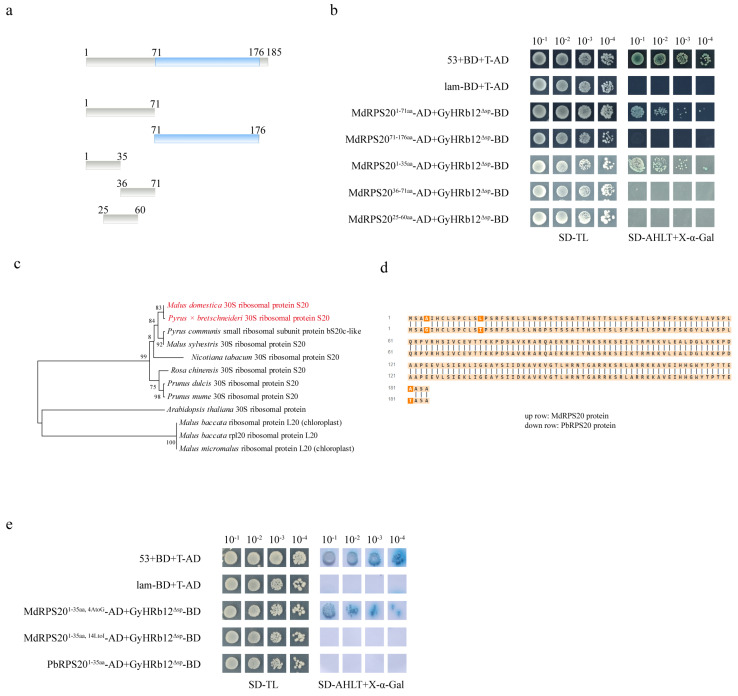
Mapping the interaction domains and key residues between GyHRb12 and MdRPS20. (**a**) Regions of MdRPS20 used to construct pGADT7 vectors of different lengths. (**b**) Interaction results between various MdRPS20 fragments and GyHRb12. (**c**) Maximum likelihood evolutionary tree of closely related species, with L20 used as the outgroup. (**d**) Protein sequence alignment between MdRPS20 and PbRPS20. (**e**) Y2H assay results of amino acid mutants of MdRPS20 with GyHRb12. In both (**b**,**e**) assays, yeast strains co-carrying constructs GyHRb12 (ΔSP)-pGBKT7 (BD) and different regions of MdRPS20-pGADT7 (AD) were grown on SD media-TL, SD-AHLT, and SD-LWHA with X-α-gal. 53-BD/T-AD was used as the positive control.

To determine whether GyHRb12 modulates MdRPS20 function via its S20 domain, a yeast two-hybrid (Y2H) assay was performed. Specifically, two truncated MdRPS20 constructs were generated (Figure 4a), MdRPS20^1–71aa^ (non-domain region) and MdRPS20^71–176aa^ (S20 domain region). The Y2H assays confirmed reliability, as the positive control (53 + T) grew normally on selective medium, while the negative control (Lam + T) failed to grow. Crucially, GyHRb12^Δsp^ interacted robustly with MdRPS20^1–71aa^ on SD/-Leu-Trp-His-Ade selection medium, whereas no yeast colonies were observed when MdRPS20^71–176aa^ was co-expressed with GyHRb12^Δsp^ (Figure 4b). To delineate the interaction domain within MdRPS20^1–71aa^, three sub-fragments were tested: MdRPS20^1–71aa^ (N-terminal) exhibited strong interaction (Figure 4a,b); conversely, MdRPS20^36–71aa^ (C-terminal) and MdRPS20^25–60aa^ (mid-region) showed no binding (Figure 4a,b), indicating that GyHRb12^Δsp^ targets the N-terminal residues 1–35 aa to influence MdRPS20 activity.

Despite sharing juniper as telial hosts, *G. yamadae* and *G. asiaticum* exhibit host specificity: the basidiospores of *G. asiaticum* infect *Pyrus* spp., while *G. yamadae* targets *Malus* spp. Multiple sequence alignment of *Rosaceae* RPS20 proteins showed that MdRPS20 and PbRPS20 from *Pyrus* × *bretschneideri* shared the highest sequence similarity (94.7%, Figure 4c), differing by only two amino acids in the N-terminal region (Figure 4d). To test whether GyHRb12 interacts with PbRPS20, three pGADT7 vectors with PbRPS20^1–35aa^, MdRPS20^1–35aa^ (4A→G), MdRPS20^1–35aa^ (14L→I) protein were generated. Strikingly, only MdRPS20^1–35aa^ (4A→G) interacted with GyHRb12^Δsp^ on SD/-Leu-Trp-His-Ade selection medium (Figure 4e), demonstrating that leucine at position 14 of MdRPS20 was indispensable for effector recognition and affirming GyHRb12 as a host-specific virulence factor.

### 2.5. GyHRb12 Might Modulate Ribosome Abundance in Apple Leaves

**Figure 5:**
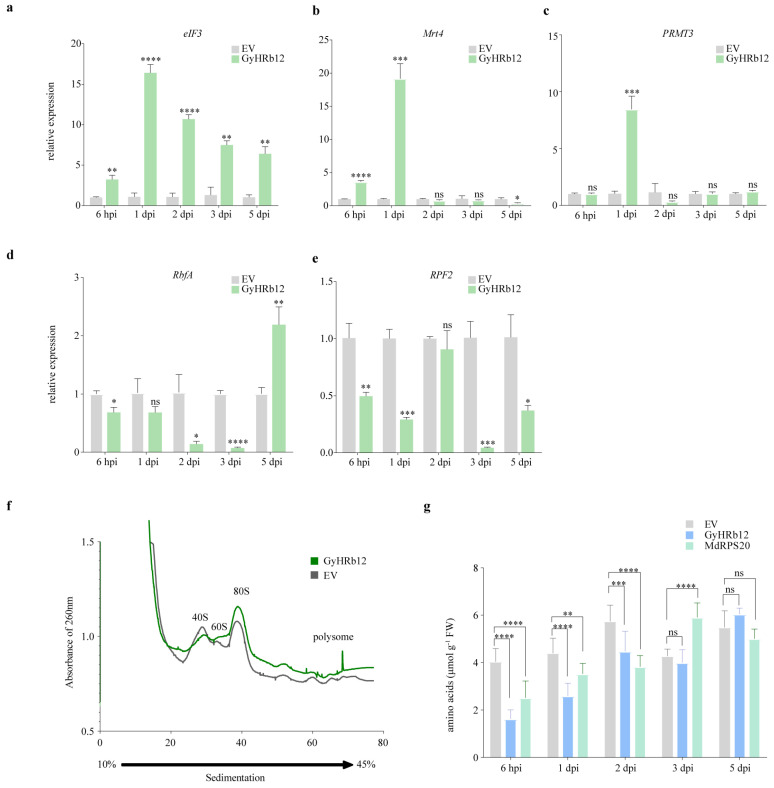
GyHRb12 is involved in ribosome biogenesis and protein synthesis in apple leaves. (**a**–**e**) Relative expression of ribosome-related protein genes (*eIF3*, *Mrt4*, *PRMT3*, *RbfA* and *RPF2*) by qRT-PCR. Significant differences compared to the EV were determined using Student’s *t*-test. (**f**) Ribosome profiling analysis in GyHRb12-expressing leaves compared with the EV control. Three major absorption peaks represent 40S, 60S ribosomal subunits, and the 80S ribosome. (**g**) Amino acid content analysis showing reduced levels of free amino acids in GyHRb12-overexpressing leaves. Values are presented as ±SD and different lowercase letters above the error bars indicate significant difference at *p* < 0.05 level as determined by one-way ANOVA with Tukey’s multiple comparisons test. * *p* < 0.05, ** *p* < 0.01, *** *p* < 0.001, **** *p* < 0.0001, and ns was not significant.

Given that MdRPS20 is a ribosomal protein and that GyHRb12 primarily interfered with protein translation in apple leaves, it is possible that GyHRb12 may consequently affect ribosomal function within the host plant. To further examine this, proteins associated with ribosome synthesis were selected (Table S5) and their transcript levels were measured by qRT-PCR (Figure 5a–e). The results were consistent with the proteomics data, showing that *eIF3*, *Mrt4* and *PRMT3* were up-regulated. Notably, while *RbfA* transcript levels decreased, its protein abundance increased, suggesting that GyHRb12 modulates ribosome biogenesis through post-transcriptional mechanisms or selective translation.

Ribosome profiling revealed an increase in both ribosomal subunits and polysomes in GyHRb12-expressing leaves compared to the EV control (Figure 5f). The non-polysome and polysome fractions in the GyHRb12 group were 220.1 and 35.8, respectively, compared with 245.1 and 35.0 in the EV group. The corresponding P/non-P ratios (0.163 vs. 0.143) suggested that GyHRb12 overexpression enhanced translational activity in apple leaves. Furthermore, the contents of amino acids were markedly depleted in GyHRb12/MdRPS20-overexpressing leaves (Figure 5g), likely reflecting accelerated protein synthesis and heightened metabolic demand. Taken together, these observations were consistent with a model in which GyHRb12 expression is associated with enhanced ribosome accumulation and translational activity in apple leaves.

## 3. Discussion

Abnormal ribosomal function is closely associated with ribosomal stress across animals and plants. In animals, hyperactivation of ribosome biogenesis drives cancer progression [37] through increasing translational capacity [38]. Moreover, RPS20 mutations have been associated with colorectal cancer predisposition [39,40]. In plants, disruption of RPS20 is also associated with defective growth phenotypes [24,25]. Notably, parallel mechanisms of ribosomal stress have been observed in plants, where ANAC082 functions as the plant counterpart of the animal p53 ribosomal stress pathway [41], highlighting the conservation of ribosomal stress signaling. Consequently, Pi23226-induced nucleolar stress was found in *N. benthamiana* [12]. At the immune level, RPS4 expression is induced by infection with *Xanthomonas oryzae* pv. *oryzae* or *Rhi-zoctonia solani*, implying a potential role in mediating responses to biotic stress [42]. Similarly, RPS15 and RPS19 demonstrate direct antimicrobial activity in amphioxus [43,44]. The present study indicates that MdRPS20 attenuates specific immune responses in apple leaves (Figure 3), contrasting with typical resistance activation. Significantly, the expression of MdRPS20 was consistent with that of GyHRb12 (Figures S1 and S2), suggesting that MdRPS20 might exert regulatory functions in disease resistance and serve as a direct target for manipulation by *G. yamadae*. Unfortunately, the functional contribution of GyHRb12-induced MdRPS20 expression to resistance remains unverified due to the unavailability of stably transformed apple seedlings. Together, these observations highlight the significance of ribosome function in plant disease resistance. Abnormal ribosome responses triggered by rust fungus parasitism in plants share mechanistic similarities with ribosomal stress responses in animal systems. Consequently, ribosomes should be considered a critical component in future studies of pathogen-host interactions.

Conserved domains often provide important insights into the potential functions of effectors [7,45]. Nevertheless, the majority of candidate effectors identified in rust fungi lack discernible conserved domains or annotation motifs, considerably impeding their functional characterization [32,34,46]. Therefore, using an efficient system to verify the function of these uncharacterized effectors is of considerable importance. Heterologous expression systems, such as *N. benthamiana*, have therefore been widely used to assess rust effector activity; indeed, several rust effectors, including Pst_23 and Pst_8713, have been reported to suppress immune responses in this system [9,47]. In the present study, although GyHRb12 lacks recognizable conserved domains or motifs (Figure 1a) and fails to modulate PCD responses in tobacco leaves [36], this effector significantly attenuates PCD while modulating protein biosynthesis in its primary host apple leaves (Figure 1 and Figure 2). This functional disparity underscores the constraints of heterologous assays and suggests that certain effectors may exert host-specific functions that cannot be captured outside their natural context. Future research should thus prioritize functional validation of candidate effectors within their original host plants, with particular emphasis on those devoid of conserved domains.

The effector proteins may exert dual functions of regulating host immune responses while simultaneously promoting nutrient transport to the pathogen. Previous studies have demonstrated that haustoria-secreted transporters play crucial roles in nitrogen acquisition during infection. For instance, TaAMT2;3a functions as an ammonium transporter that facilitates nitrogen uptake from host cells [48]. The cystine transporter PstCYN1 also serves this role. It acquires host-derived cysteine for fungal nutrition and, by promoting its conversion to cystine, concurrently scavenges reactive oxygen species (ROS). Thus, PstCYN1 performs the dual function of nutrient acquisition and host immune suppression [49]. The observed promotion of amino acid consumption (Figure 5g) and protein synthesis (Figure 2c) by GyHRb12 may represent preparatory events for nitrogen mobilization, potentially increasing the availability of host-derived nitrogen for fungal uptake. Collectively, by priming host metabolism for nitrogen mobilization, GyHRb12 may concurrently facilitate nutrient acquisition and create an environment conducive to infection, serving dual roles in pathogenicity.

The specific interaction between GyHRb12 and *M. domestica* may indicate a potential mechanism underlying host selection by *G. yamadae.* Considering the high conservation of ribosomal proteins, the CDS of MdRPS20 and PbRPS20 (*Pyrus* × *bretschneideri*) were compared and only two amino acid differences within the N-terminal region (1–35aa) were found (Figure 4a,b). Our findings indicate that Leu14 is critical for GyHRb12-MdRPS20 recognition, as mutation to isoleucine completely abolished their interaction in our in vivo assays (Figure 4e). From the pathogen perspective, this structural difference may account for the inability of GyHRb12 to function in pear leaves. This observation is consistent with a previously reported co-adaptation mechanism involving PstGTA1 and TaSIG in a wheat–rust pathosystem [8]. Although a functional counterpart of GyHRb12 may operate in *G. asiaticum* to regulate protein biosynthesis in pear leaves, transcriptomic data from *G. asiaticum* did not retrieve a sequence homolog in our screening. Collectively, the GyHRb12-MdRPS20 interaction may represent a potential example of adaptive molecular co-evolution between rust pathogens and plants, though its precise role in non-host resistance awaits validation in a stable genetic system.

Targeting specific domains of host proteins may represent one of the strategies for effector-mediated virulence modulation [50,51]. For instance, FolSvp2 targets the SIPR1 N-terminal region to inhibit its entry into plant cells and prevents FolSvp2-mediated translocation of SlISP, abolishing its contribution to fungal virulence [52]. Similarly, Pt_21 interacts with TaTLP1 in the apoplast, requiring Cys71 within its N-terminus, though dispensable for TaTLP1 function [53]. GyHRb12 was found to interact specifically with the N-terminal region of MdRPS20 (Figure 4a,b), and BiFC assays (Figure 3b) showed that GyHRb12 co-localizes with MdRPS20 in the cytoplasm. These results suggest that GyHRb12 may promote the relocalization of MdRPS20 through interaction with its chloroplast transit peptide. Collectively, these findings highlight a previously unrecognized strategy by which rust fungal effectors may interfere with host ribosomal function to fine-tune cellular homeostasis and immunity. Future investigations should validate MdRPS20 accumulation dynamics across compartments to determine if GyHRb12 promotes its cytoplasmic translocation for ribosome assembly.

MdRPS20 is predicted to be a component of chloroplast 30S ribosomal subunit and may contain both a chloroplast transit peptide and a nuclear localization signal (Figure S3). Although chloroplast 30S ribosomal proteins are generally synthesized in the cytosol and imported directly into chloroplasts without nucleus accumulation [54], MdRPS20 was predominantly observed in the nucleus and cytosol in our subcellular localization assays (Figure 3e), with no chloroplast signal detected. This discrepancy may reflect an evolutionary remodeling of MdRPS20 localization in woody plants [55,56]. Alternatively, it could also result from the N-terminal GFP fusion in the pBinGFP vector masking the chloroplast transit peptide, thereby interfering with proper chloroplast targeting [57].

**Figure 6:**
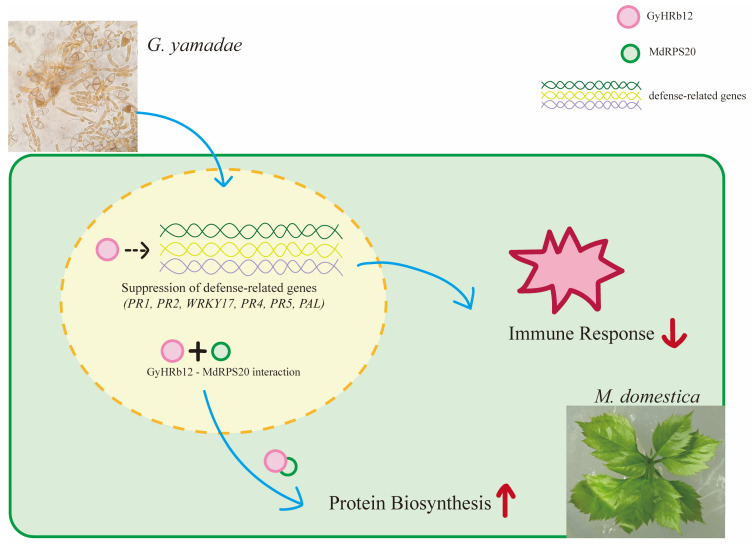
GyHRb12 interacts with the ribosomal protein MdRPS20 to enhance protein biosynthesis (red upward arrow), while simultaneously suppressing the expression of defense-related genes (*PR1*, *PR2*, *WRKY17*, *PR4*, *PR5*, *PAL*) through an unknown mechanism (black arrow), thereby attenuating the host immune response (red downward arrow).

Consequently, an interaction model was proposed as follows (Figure 6). Following haustorium formation, *G. yamadae* secretes GyHRb12 into host cells, where nuclear translocation enables interaction with MdRPS20 in both nuclear and cytoplasmic compartments. Concurrently, the expression of host defense-related genes and the activation of immune responses are suppressed. In contrast, untreated leaves exhibit successful activation of immune responses.

## 4. Materials and Methods

### 4.1. Biological Materials and Growth Conditions

Five-year-old *M. domestica* ‘Gala3’ plants were cultivated at the Beijing Forestry University (Beijing, China). Tissue-cultured ‘Gala3’ seedlings were maintained on Murashige and Skoog (MS) medium at 25 °C. *N. benthamiana* seedlings were grown in a controlled chamber under long-day conditions (16 h light/8 h dark) with day/night temperatures of 25 °C and 19 °C, respectively. *G. yamadae* galls were harvested from infected *Juniperus chinensis* located in Haidian Park (Beijing, China). *E. coli* strains DH5α and BL21 were cultured at 37 °C, *A. tumefaciens* strains GV3101 and EHA105 were cultured at 28 °C, yeast strain Y2HGold was cultured at 30 °C.

### 4.2. Western Blot Analysis

The coding sequence of GyHRb12 (without the stop codon) was fused to mCherry [58] and inserted into the pGR106 vector. Total proteins were extracted from infiltrated leaves using a plant protein extraction kit (CWBIO, Taizhou, China, CW0885M). Protein samples were resolved by SDS-PAGE and transferred onto PVDF membranes (Sigma-Aldrich Immobilon, St. Louis, MO, USA, IPVH00010) using a Trans-Blot Turbo Transfer System (Bio-Rad, Hercules, CA, USA, 1704150). Membranes were blocked with 5% non-fat dry milk prepared in Tris-buffered saline with 0.1% Tween 20 (TBST) and incubated with primary anti-mCherry (1:10,000, Abclonal, Wuhan, China, AE127) or anti-GFP (1:10,000, Abclonal, Wuhan, China, AE078) antibodies at 4 °C for 12 h. The membrane was washed five times with TBST for 5 min each before addition of secondary HRP-conjugated goat anti-rabbit IgG (1:10,000) for 1 h at room temperature. Chemiluminescent detection was subsequently conducted using ECL substrate (Abclonal, Wuhan, China, RM00021P) according to the manufacturer’s instructions.

### 4.3. Immune Response Assays in M. domestica

The CDS of GyHRb12 was amplified and inserted into pGR106 vector, which was subsequently transformed into *A. tumefaciens* GV3101. Then, the bacteria were collected and resuspended in 10 mM MgCl_2_ containing 100 μM acetosyringone to achieve a final OD600 of 0.4. The bacterial suspensions were infiltrated into the apple leaves by vacuum infiltration. Two days later, trypan blue staining was performed to visualize cell death, and the area were quantified using ImageJ (v1.54d) software [59]. H_2_O_2_ content was determined using the titanous sulfate method (Micny Biology, Suzhou, China, kit M0107), and O_2_^−^ content was measured using the hydroxylamine oxidation method (Micny Biology, Suzhou, China, kit M0114). For callose deposition analysis, leaf samples collected 4 days post-infiltration were cleared using saturated chloral hydrate solution. Following sequential treatments with 50% ethanol, 0.5 M NaOH, and 0.067 M K_2_HPO_4_, tissues were stained with 0.05% aniline blue solution overnight and visualized by fluorescence microscopy the following day and quantitative statistics of callose results were quantified using ImageJ (v1.54d) software.

### 4.4. qRT-PCR Analysis

Apple leaves were inoculated with freshly germinated basidiospores of *G. yamadae* according to the method described by Shao et al. [60,61]. Briefly, basidiospores were collected after germination and uniformly applied to the surfaces of apple leaves under controlled humidity to facilitate infection. Leaf samples were harvested at 0, 3, 6, 10, 16, 26, 36, 60, 67, and 77 days post-inoculation. Collected samples were immediately frozen in liquid nitrogen and stored at −80 °C until RNA extraction and subsequent gene expression analysis.

Total RNA was extracted according to the instructions of AFTSpin Universal Plant Fast RNA Extraction Kit (Abclonal, Wuhan, China, RK30121), followed by integrity verification through agarose gel electrophoresis and quantification with NanoDrop2000. Reverse transcription was conducted with ABScript III RT Master Mix (Abclonal, Wuhan, China, RK20429). Quantitative RT-PCR (qRT-PCR) amplification was performed with a Bio-Rad Detection system using 2× Universal SYBR Green Fast qPCR Mix (Abclonal, Wuhan, China, RK21203). Result analysis utilized the 2^−ΔΔCt^ method [62]. The specific primers used in this study are listed in Supplementary Table S6. 18S rRNA was used as the internal reference gene [63]. The resistance-associated genes were *LOX1* [64], *PR4* [65], *PR2* [66], *PR5* [67], *PR1* [68], *PAL* [69], and *WRKY17* [70]. Data are means (±SEM) from three biological replicates.

### 4.5. Proteomics Analysis

For data-independent acquisition (DIA) mass spectrometry analysis, proteins were extracted from *A. tumefaciens*-infiltrated apple leaves with three biological replicates of apple leaves. Each homogenized sample was mixed with 300 μL of 8 M urea containing 10% protease inhibitor cocktail. After centrifugation at 14,100× *g* for 20 min, the supernatant was collected for protein quantification using the Bradford assay. Aliquots of the remaining samples were preserved at −80 °C until further processing.

Protein samples were subsequently reduced with 10 mM TCEP and alkylated with 25 mM CAA at 37 °C for 30 min. After dilution with 10 mM TEAB buffer, trypsin digestion was carried out overnight at 37 °C using a 50:1 protein-to-trypsin ratio. Digestion was stopped by adjusting pH < 3 with formic acid. Peptides were desalted using C18 columns, eluted with 70% acetonitrile, lyophilized, and stored at −80 °C.

Chromatographic separation utilized a 25 cm analytical C18 column (150 μm inner diameter, 1.9 μm particle size) with a linear 65-min gradient of 8–40% solvent B (80% acetonitrile, 0.1% formic acid) at a constant flow rate of 600 nL/min. Mass spectra were acquired in DDA (“top-40”) and DIA modes on a Q Exactive HF-X (Thermo Fisher Scientific, Bremen, Germany).

Both DDA and DIA datasets were processed in Spectronaut (v15.7) to construct a hybrid spectral library against the *M. domestica* UniProt database (45,119 entries). During database searching, Carbamidomethylation was specified as a fixed modification, whereas variable modifications included N-terminal acetylation and methionine oxidation. A maximum of two missed cleavages were allowed. Differential expression analysis was performed using Student’s *t*-test based on normalized protein intensity values. Proteins with a fold change ≥ 1.5 or ≤1/1.5 and *p* < 0.05 were defined as differentially expressed proteins. Functional annotation and enrichment were conducted using InterProScan-5, COG, KEGG.

### 4.6. Yeast Two-Hybrid Screening

The cDNA library in pGADT7 was constructed from *G. yamadae*-infected *M. domestica* leaves. Concurrently, the coding sequence of GyHRb12^Δsp^ was inserted into pGBKT7 (BD) for bait construction. Initially, the cDNA library and pGBKT7-GyHRb12^Δsp^ was co-transformed into yeast strain Y2Hgold, and the yeast strain was incubated on SD/-TL (lacking leucine and tryptophan), SD/-THLA with X-α-gal (lacking leucine, tryptophan, histidine, and adenine) media to screen for proteins potentially interacting with GyHRb12. Subsequently, MdRPS20 and its truncated or mutated versions were cloned into pGADT7 (AD) vector to be co-transformed with GyHRb12-pGBKT7 into Y2Hgold under identical incubation parameters to validate protein-protein interactions.

### 4.7. GST Pull-Down Assay

For the construction of His-GyHRb12 and GST-MdRPS20 recombinant proteins, the GyHRb12^Δsp^ coding sequence was inserted into the pET28a vector, whereas MdRPS20 was cloned into the pGEX6p-1 using specific primers (Table S6). Both vectors were transformed into *E. coli* BL21 cells to facilitate protein expression. Protein expression was induced with 0.3 mM IPTG at 16 °C in LB cultures. His-GyHRb12 and MdRPS20-GST proteins were purified using Ni-NTA Agarose (QIAGEN, Shanghai, China, No. 30230), while MdRPS20-GST was isolated using Glutathione Sepharose™ 4B beads (Cytiva, Shanghai, China, 170756). Following purification, the recombinant proteins were separately eluted using His Elution Buffer and GST Elution Buffer, with expression confirmed through Western blot analysis. For the pull-down assay, purified His-GyHRb12 protein was incubated with MdRPS20-GST pre-bound to GST beads at 4 °C for 4 h. After removing the supernatant, the beads were washed several times with GST wash buffer, and the bound proteins were resuspended in 5× protein loading buffer for Western blot analysis. Detection involved anti-GST-Tag antibody (Abmart, Shanghai, China, M20007), anti-His-Tag antibody (Abmart, Shanghai, China, M20001), and IRDye 800CW Goat anti-Mouse IgG (Licor, Lincoln, NE, USA, 925-32210) antibodies. Experimental results were documented using an Odyssey CLx Imager (Licor, Lincoln, NE, USA).

### 4.8. Fluorescence Confocal Microscopy

The CDS of GyHRb12^Δsp^ and MdRPS20 were cloned into the BiFC vectors to generate pDEST-^GW^VYNE- GyHRb12^Δsp^ and pDEST-^GW^VYCE-MdRPS20, respectively. Subcellular localization of MdRPS20 was determined using the pBinGFP2 vector. For BiFC experiments, according to the manufacturer’s recommendations for the Gateway kit (Invitrogen, Shanghai, China, 12536-017 and 12535-035). Subcellular localization assay and BiFC assay constructed vectors were transformed into *A. tumefaciens* GV3101. *N. benthamiana* leaves were infiltrated with the bacterial suspension using a needleless syringe. After 48 h, fluorescence signals were observed using a Leica SP8 LIGHTNING confocal microscope (Leica Microsystems, Wetzlar, Germany). The primers used in this study are listed in Table S6.

### 4.9. Polysome Profile

Fresh tissue samples were initially rinsed with ultrapure water before surface drying with absorbent paper to eliminate contaminants. Subsequently, materials were rapidly minced and flash-frozen in liquid nitrogen. The frozen tissue was ground to a fine powder in a mortar precooled with liquid nitrogen. Following transfer of the ground powder to a centrifuge tube, lysis buffer was added, and the mixture was incubated on ice for lysis. After centrifugation, the supernatant was carefully stratified onto a preformed sucrose gradient (10% to 45%). Gradients were balanced and subjected to ultracentrifugation at 4 °C. Fractions were collected using an automatic fraction collector operating at 1.5 mL/min with UV detection at 260 nm. Resulting concentration profiles were generated through continuous absorbance measurement. Collected fractions were assigned numbers; samples corresponding to specific peaks identified in the profile plot were selected based on their elution time (minutes) for further separation and labeling.

### 4.10. Amino Acid Content Analysis

Apple leaves transiently transformed with GyHRb12-PBI121 or MdRPS20-PBI121 were harvested at 3 days post-transformation. After grinding in liquid nitrogen, the free amino acid content was measured with the ninhydrin colorimetric method following the manufacturer’s protocol (Micny Biology, Suzhou, China, M0501).

### 4.11. Bioinformatics Analyses

Database searches were performed using NCBI (https://www.ncbi.nlm.nih.gov) and Uniprot database (https://www.uniprot.org) for homolog identification. Conserved domain prediction was conducted using NCBI and SMART (https://smart.embl.de/, accessed on 10 September 2024). The sequence motif was predicted by MEME [71] (https://meme-suite.org/meme/, accessed on 10 September 2024). InterPro (https://www.ebi.ac.uk/interpro/, accessed on 10 September 2024) was used to predict the functional annotation of MdRPS20. TargetP 2.0 (https://www.healthtech.dtu.dk/, accessed on 6 March 2026) was used to predict protein transit peptides. LOCALIZER (https://localizer.csiro.au/, accessed on 6 March 2026) was used to predict subcellular localization.

### 4.12. Statistical Analysis

All experimental procedures were replicated at least three times. Statistical significance was determined using GraphPad Prism v.8.0.2.

## Data Availability

The original contributions presented in this study are included in the article and Supplementary Materials. Further inquiries can be directed to the corresponding author.

## Supplementary Materials

The following supporting information can be downloaded at: https://www.mdpi.com/article/10.3390/ijms27072970/s1.
