# Supplementary material for: Gymnosporangium yamadae Effector GyHRb12 Targets the Host Ribosomal Protein MdRPS20 to Enhance Translation and Suppress Immunity of Apple Leaves
Source: Int J Mol Sci. 2026 Mar 25;27(7):2970. doi: 10.3390/ijms27072970 (PMC13072964; doi:10.3390/ijms27072970)
Supplement: Supplementary file 1 [file ijms-27-02970-s001.zip › Supplementary figureS1.pdf]

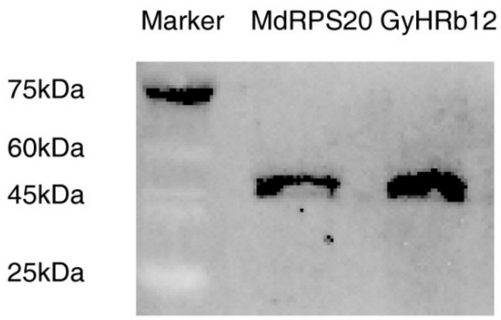

Supplementary Figure S1. The expression levels of proteins MdRPS20 and GyHRb12 were detected by Western blot. The protein levels of MdRPS20-mCherry and GyHRb12-mCherry detected by western blotting. Total proteins were immunoblotted with anti-mCherry antibodies. Experiments were repeated three times with similar results.
