# Supplementary material for: Gymnosporangium yamadae Effector GyHRb12 Targets the Host Ribosomal Protein MdRPS20 to Enhance Translation and Suppress Immunity of Apple Leaves
Source: Int J Mol Sci. 2026 Mar 25;27(7):2970. doi: 10.3390/ijms27072970 (PMC13072964; doi:10.3390/ijms27072970)
Supplement: Supplementary file 1 [file ijms-27-02970-s001.zip › Supplementary figureS2.pdf]

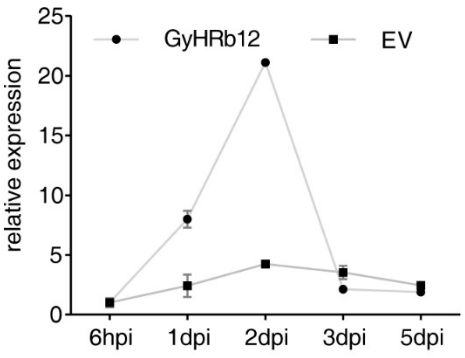

Supplementary Figure S2. GyHRb12 relative expression in apple leaves. The relative expression of *GUS* in EV and *GyHRb12* in the GyHRb12 group was detected in apple leaves after GV3101 infiltration using reverse transcription-quantitative PCR assay, with expression at 6 hours post-infiltration (hpi) set as the reference for each group. Results were presented as the mean ±SD of three replicates.
