# Supplementary material for: Gymnosporangium yamadae Effector GyHRb12 Targets the Host Ribosomal Protein MdRPS20 to Enhance Translation and Suppress Immunity of Apple Leaves
Source: Int J Mol Sci. 2026 Mar 25;27(7):2970. doi: 10.3390/ijms27072970 (PMC13072964; doi:10.3390/ijms27072970)
Supplement: Supplementary file 1 [file ijms-27-02970-s001.zip › Supplementary figureS3.pdf]

a

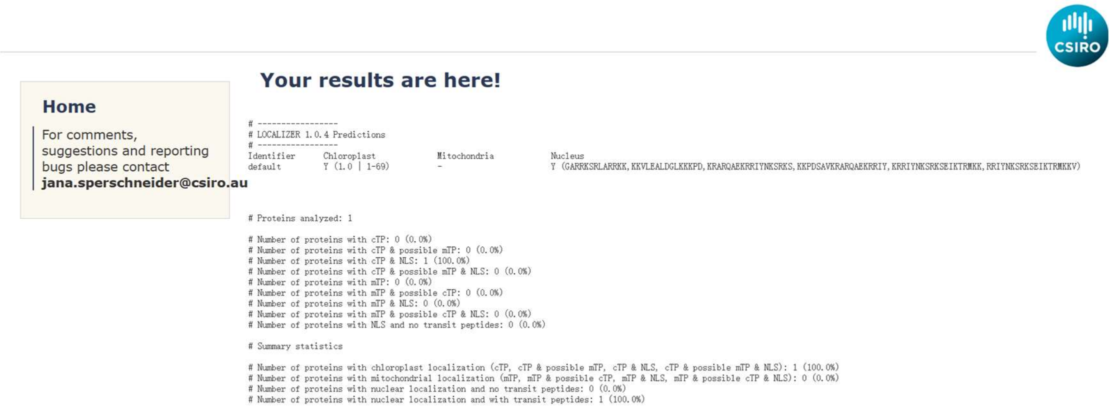

b

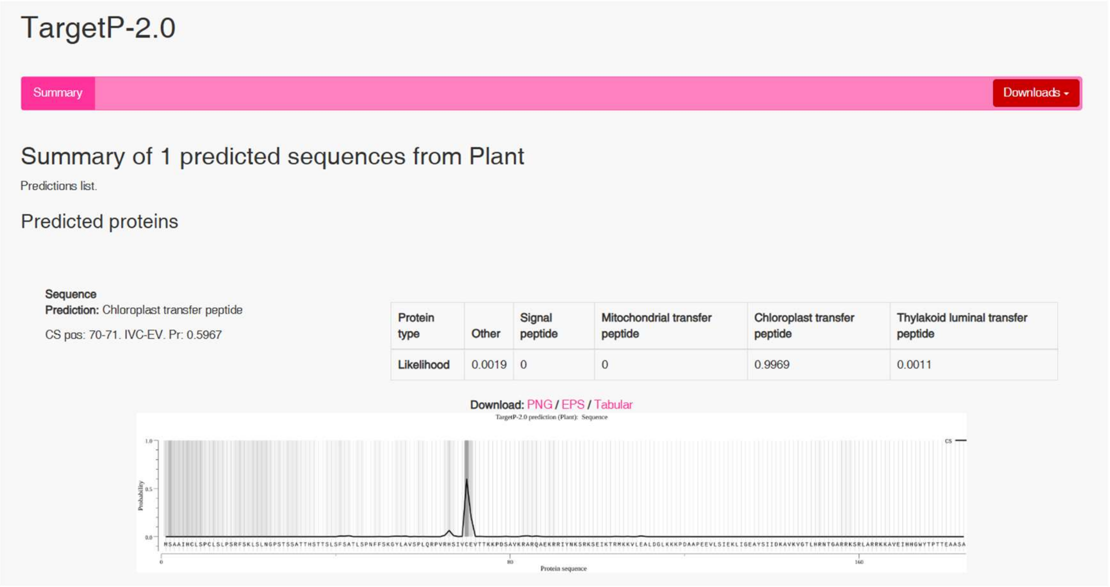

Figure S3. Subcellular localization and transit peptide prediction of MdRPS20. a Subcellular localization of MdRPS20 predicted by LOCALIZER. **b** Transfer peptide prediction of MdRPS20 using TargetP 2.0.
